# Physiochemical characterization and systematic investigation of metals extraction from fly and bottom ashes produced from municipal solid waste

**DOI:** 10.1371/journal.pone.0239412

**Published:** 2020-10-22

**Authors:** Mohammad A. Al-Ghouti, Mariam Khan, Mustafa S. Nasser, Khalid Al Saad, O. O. N. Ee Heng

**Affiliations:** 1 Department of Biological and Environmental Sciences, College of Arts and Sciences, Doha, Qatar University, State of Qatar, Western Asia; 2 Gas Processing Center, College of Engineering, Qatar University, Doha, State of Qatar, Western Asia; 3 Department of Chemistry and Earth Sciences, College of Arts and Sciences, Qatar University, Doha, State of Qatar, Western Asia; 4 Domestic Solid Waste Management Centre (DSWMC), Doha, State of Qatar, Western Asia; Indian Institute of Technology Patna, INDIA

## Abstract

Incineration has emerged as one of the acceptable ways to treat municipal solid waste (MSW) due to its potential in reducing the mass and volume of the waste. However, it produces two major by-product residues, namely MSW-bottom ash (MSW-BA) and MSW-fly ash (MSW-FA). These residues have gained great attention to their hazardous nature and potential to be reused and recycled. In this paper, the physicochemical characterizations of the MSW-BA and the MSW-FA were performed, followed by a systematic investigation of metals extraction from MSW-BA and MSW-FA. Various extracting agents were used to investigate the possibility to extract 21 metals including cadmium (Cd), vanadium (V), chromium (Cr), and lead (Pb). It was revealed that some metals were present in a high amount in the MSW-BA while other metals were higher in the MSW-FA. Moreover, the energy-dispersive X-ray spectroscopy results revealed that the MSW-BA was dominated by oxygen (O) 55.4 ±0.6 wt%, silicon (Si) 22.5 ±0.3 wt%, and calcium (Ca) 18.5 ±0.2 wt%. On the other hand, the MSW-FA was enriched with Ca 45.2 ±0.5 wt%, and O 40.3 ±0.4 wt%. From the scanning electron microscopy, the MSW-BA was observed as flaky with an irregular surface that consisted of large pores, while, the MSW-FA was present as agglomerated particles and had a bimodal distribution. Moreover, Fourier transform infrared spectroscopy revealed that Al-Fe-OH, Al-Al-OH, Si-O, C-O, and C-H were some of the major functional groups present in the ashes. The F-tests concluded that the metal extraction from the MSW-BA and MSW-FA were significantly affected by the acid type. it is concluded that nitric acid and phosphoric acid were the best-suited acid for the MSW-BA while sulfuric acid and phosphoric acid for the MSW-FA. More than 11 wt% of Cd and 9 wt% of Cu were extracted from MSW-BA while 6 wt% of Pb and 4.5 wt% of V were extracted from the MSW-FA. The present methodology is an interesting development in metal extraction from the MSW-BA and the MSW-FA, which can develop in a cost-effective and sustainable option to utilize MSW.

## 1. Introduction

The management of manuscript solid waste (MSW) has become one of the major issues around the world and is, therefore, a key concern for municipal societies. A successful MSW management should execute proper management control, including planning, assortment, transportation, treatment, extraction, recovery recycling, and disposal. One of the most predominant practices used to treat MSW is incineration, which uses innovative treatment solutions to produce green energy and reduce the volume and mass of MSW up to 80% and 90% respectively [1,2,3]. However, the two major by-product residues from incineration namely, MSW-bottom ash (MSW-BA) and MSW-fly ash (MSW-FA) [4] have raised concerns regarding the well-being of the environment and living organisms. MSW-BA is a glassy-type material collected from the combustion chamber, which contains metals and salts that could be used as raw materials [3,5]. MSW-FA, on the other hand, is a fine hazardous by-product which poses challenging environmental issues, due to the presence of leachable metals and toxic organic substances, such as dioxins, furans, and PAHs [4]. The enormous amount of MSW-BA and MSW-FA that are generated from incineration plants are laden with various metals including Cobalt (Co), Copper (Cu), Chromium (Cr), Cadmium (Cd), Iron (Fe), Manganese (Mn), Magnesium (Mg), Barium (Ba), Lead (Pb), Aluminium (Al), Vanadium (V), and Zinc (Zn) that are dumped in various landfills resulting in loss of marketable resources. Thus, extracting and recovering these metals will not only preserve natural resources but also transform the hazardous ashes into their inert form.

Waste re-utilization is a promising way towards developing an environmentally friendly and cost-effective products [5–14]. Incinerated MSW residues can be described as unexplored residues that are rich with minerals and metals. There are many technologies present that are used to extract metals from ashes, such as hydrothermal [6], adsorption [15], microwave digestion, and ultrasonication [16]. However, these technologies have high operating costs and require long leaching time. Solvent extraction is one of the common techniques practiced, to extract elements from various sediments. However, some studies now believe that the European Community Bureau of Reference (BCR) is not the ideal solution for metal extraction as minerals/elements react differently to chemical agents [7] and can also result in an incomplete dissociation of metals [8]. BCR has been reported to undermining the correct quantity of acid dissolved metals with those bounded with carbonate phase in sediments rich in carbonates [9]. However, studies claim, the recovery of metals from acid solution has the lowest environmental impact [10].

Metal extraction using different extraction agents is one of the best techniques used to investigate the metal-binding mechanisms within solid residues [11]. It is worth pointing out, that even though the MSW-BA represents the major fraction of the incinerated MSW by-products, no systematic investigations have been till date been performed [12,13]. Table 1 shows the different experimental conditions that have been done on various solid wastes to extract metals [14,17–23].

**Table 1:** Different experimental conditions used for metal extraction from solid wastes.

Sample type	Chemicals and procedure	Reference
**Coal fly ash**	400 mg (LiBO_2_) fused. Then digested with 5% HNO_3_ at low heat	14
**Coal Ash**	Mixture of 5-mL EDA and 5-ml of NMP. Followed by microwave treatment, with the required amount of NMP.	17
**Volcanic ash soil**	Acidic mixture of 6 mL HNO_3_ + 0.5 mL HBF_4_	18
**Mercury from fluorescent lamps**	3HCl:6HNO_3_	19
**Marine Sediment**	2 mL concentrated HNO_3_ + 6 mL concentrated HCl	20
**Municipal solid waste**	20 mL of 3.75% chelating agent was added and then 3 M of HCl was used to adjust the pH.	21
**Municipal solid waste–fly ash**	40 mL of 0.1 M citric acid leaching solution was added. The liquid to solid ratio followed was 20:1. After the leaching test, the final pH was determined and then centrifuged again and the solution was acidified using HNO_3_	22
**MSW-BA**	The metal specification followed a sequential extraction procedure (SEP)	23

For a small country like Qatar, the generation of the MSW is as high as 4,500 tons per day [3,23–25] and the principal method of disposal is landfilling. The enormous amount of MSW generated can primarily be due to the rapid urbanization, increase in living standards, and high buying power due to the rapid economic expansion, coupled with a lack of awareness for sustainable waste management practices. The composition of the Qatari MSW consists mainly of organic materials, while the rest consists of paper, glass, plastics, and metals. The amount of metal present in Qatar’s MSW is very high (9 wt%) [16], which is higher than the USA (8 wt%) [26], Germany (5 wt%) [26], and Russia (4.7 wt%) [26]. Recovering valuable metals from the ashes will not only be beneficial for the economy but also create environmental stabilization.

In this study, the physicochemical characterizations of the MSW-BA and MSW-FA from one of Qatar’s MSW incineration plants were studied. Also, the best suited extracting agents were determined by using several agents, namely (i) inorganic salt extracting agents, including sodium acetate, diammonium ethylenediaminetetraacetate (EDTA) (ethylenediaminetetraacetic acid diammonium salt), ammonium oxalate, ammonium sulfate, ammonium nitrate, (ii) acidic extracting agents such as nitric acid, phosphoric acid, perchloric acid, and sulfuric acid, and (iii) alkaline extracting agents such as sodium carbonate and sodium hydroxide to extract metals from MSW-BA and MSW-FA.

## 2. Methodology

### 2.1. Sample collection and preparation

Because of the complexity of the MSW-BA and MSW-FA, replicate samples were collected at different months for one year. The MSW-BA and MSW-FA were freshly collected in representative 5 kg samples from a local incinerator (Qatar Company: Domestic Solid Waste Management Centre (DSWMC)–Doha, (State of Qatar). The permission of the sample collection was already granted by the Company. We confirm that the field studies did not involve endangered protected species. The sample was dried at 100°C for 24 h, ground at different particle sizes (0.350–0.250 μm, 0.250–0.125 μm, 0.125–0.063 μm, and >0.063 μm), and sieved through a standard sieve to obtain homogenized ashes. Then, the sample was kept in a clean and isolated glass bottle. The particle size used in this study was 0.125–0.250 μm to carry out all the analytical work.

### 2.2. Elemental composition of the MSW-BA and MSW-FA

Total acid digestion was performed to determine the total metal content present in the MSW ashes according to USEPA SW846 Method 3050B (Acid Digestion of Sediments, Sludges, and Soils). 1 g of dried powdered ash was carefully digested at 90°C in Teflon^®^ using a sequence of mineral acids (nitric acid, hydrogen peroxide, and hydrochloric acid) [27] followed by analysis using an inductively coupled plasma optical emission spectrometer (ICP-OES) (Perkin-Elmer Optima 3000V, or Shimadzu ICPS-7510 Sequential Plasma Spectrometer, Japan). The moisture content analyses of the MSW-BA and MSW-FA were performed by heating the ashes to 100°C for 24 h. The pH of the solution (pH_solution_) was determined by preparing aqueous extracts at 1:1 ratios of solid: distilled water (w/v) and mixing it at room temperature for 24 h using a stirrer. The samples were then filtered using a 0.45 μm pore membrane and the filtrate pH was recorded using a pH meter (HQ440d multimeter).

#### 2.2.1. Ion chromatography (IC)

Ion chromatography (IC) (METROHM model 850 professional) was used to determine the ion content presents in the MSW-BA and the MSW-FA.

### 2.3. Physiochemical characterizations of the collected ashes

#### 2.3.1. X-ray Diffraction (XRD)

The determination of the mineralogical composition of the MSW-BA and the MSW-FA was identified by powder X-ray diffraction (XRD) (PANalytical Empyrean/Netherland). The scan was run from 5 to 85 (2-theta-scale).

#### 2.3.2. Fourier transform infrared spectroscopy (FTIR)

FTIR was used to identify the functional group on the surface of the MSW-BA and the MSW-FA using the PerkinElmer 400 Spectrum instrument using UATR (Universal Attenuated Total Reflectance) and spectra ranged from 400 to 4000 cm^−1^. The pellets were prepared by mixing 1 mg of powdered samples with 300 mg of potassium bromide.

#### 2.3.3. Scanning electron microscopy- energy-dispersive X-ray spectroscope (SEM-EDX)

Scanning electron microscopy- energy-dispersive X-ray spectroscope (SEM-EDX) (Nova™ Nano SEM 50 Series, FEI Company) was used to determine the morphology of the MSW-BA and the MSW-FA.

#### 2.3.4. Particle size distribution, surface area, pore radius, and pore volume

Laser diffraction (Malvern Mastersizer 3000 Particle Size Analyzer) was used to determine the particle size distribution for each fraction. The median of each particle size is denoted by D_50_, which is an important aspect as it tells where the 50% of the cumulative particle size lies. While D_w_ gives a better understanding of the particle size on the volume occupied, and particle distribution (P_D_) explains how particle size is similar to the particle distribution. D_w_ and P_D_ were calculated by using (Eqs 1–3).

$$\bar{D_{w}}=\frac{\sum N_{i}D_{i}^{4}}{\sum N_{i}D_{i}^{3}}$$ (1)

$$\bar{D_{n}}=\frac{\sum N_{i}D_{i}}{\sum N_{i}}$$ (2)

$$P_{D}=\frac{\bar{D_{w}}}{\bar{D_{n}}}$$ (3)

Where $\bar{D_{n}}$ is the number-average diameter, $\bar{D_{w}}$ is the weight-average diameter, *N*_*i*_ is the number of particles at the *i* th class in the size-distribution histogram.

For the Brunauer-Emmett-Teller (BET) surface area, pore radius and pore volume, the surface area analyzer (Quantachrome Corporation, Nova 3000) was used.

### 2.4. Metals extraction using different extraction media

**Fig 1:**
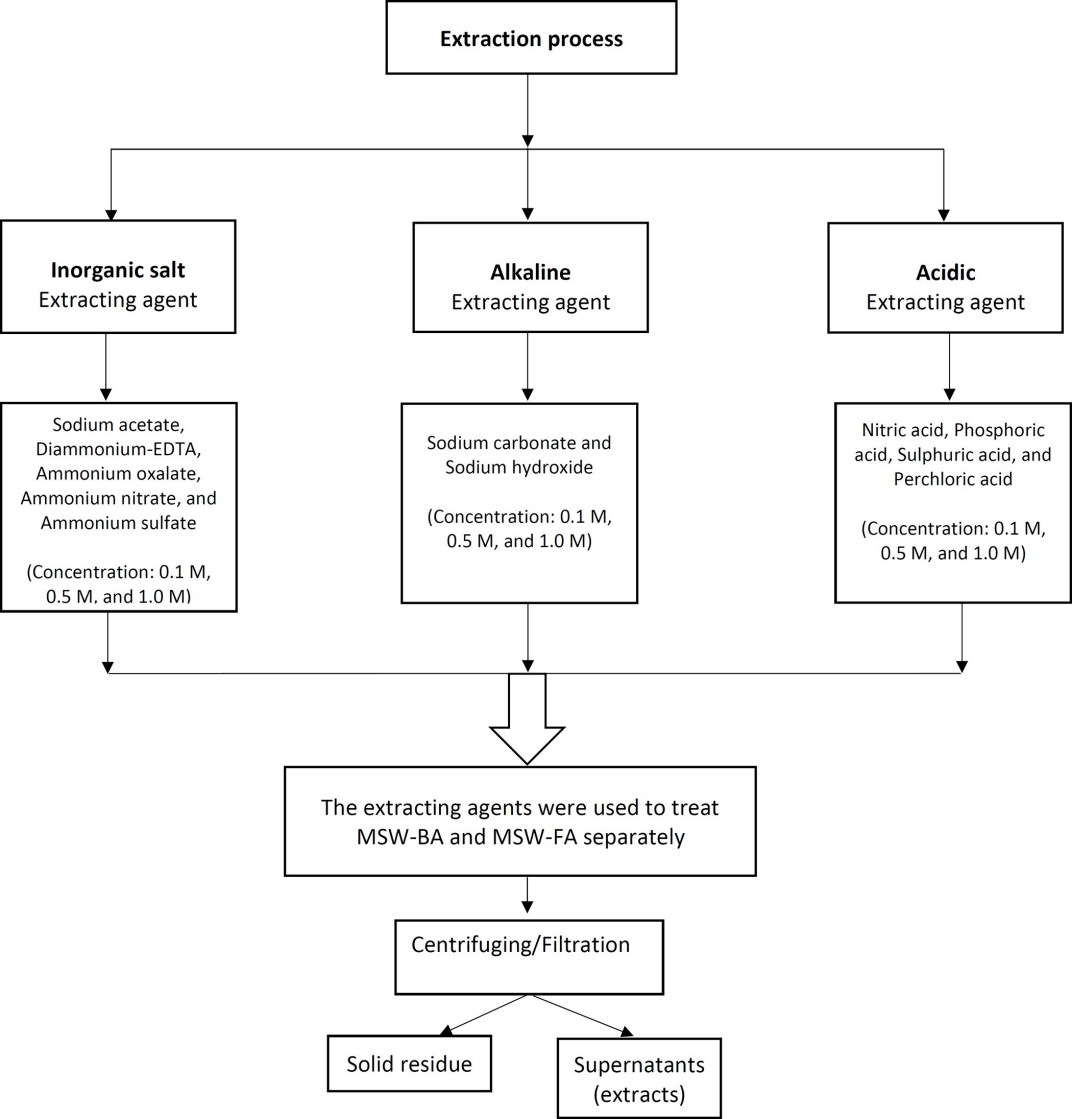
Scheme 1. Suggested extraction procedures adopted in this current study.

The metal extraction from the MSW-BA and the MSW-FA was carried out using three types of extraction media: (i) inorganic salt, (ii) acidic extracting agents, and (iii) basic extracting agents. Two influencing factors were taken into account, including the extracting agent concentration (namely 0.1 M, 0.5 M, and 1.0 M) and the solution temperature (namely 25°C and 60°C). 1.0 M stock solution was prepared for all the extracting agents and then diluted to 0.1 M and 0.5 M, The “liquid to solid” “L/S” ratio was kept constant at 50 mL/0.05 g. Then the optimal combination was determined by the range analysis. Scheme 1 (Fig 1) shows the suggested extraction procedures adopted in this current study.

0.05 g of the MSW-BA and 50 mL of the prepared extracting agents as mentioned in Scheme 1 (Fig 1) were added in capped glass bottles. Based on optimum pH determination, pH 6 was set for all experiments using Jenway 370 pH meter. Moreover, the pH was adjusted using 0.1 M hydrochloric acid and 0.1 M of sodium hydroxide (NaOH). The samples were agitated in a mechanical shaker (170 rpm) for 24 h at 25°C and 60°C using a temperature-controlled shaker (Shaking Incubator, MODEL: SSI10R-2, Orbital-Shaking). The samples were then centrifuged at 4,500 rpm and further filtration through a 0.25 μm cellulose nitrate filter (47 mm diameter) (Whatman). The supernatants (extracts) were then analyzed for final metals concentration present using ICP-OES. Once the MSW-BA experiments were completed, the entire process was repeated for the MSW-FA. A total of 38 experiments was performed for each MSW-BA and MSW-FA. All experiments were run in duplicates and the average value was taken. The averaged results were reported for all cases.

### 2.5. Solubility product constant (K_sp_) for hydroxide and carbonate

The pH of the extracting agent is a key parameter that strongly affects the extraction efficiency of metal from the ash. The possible extraction mechanisms that may occur at neutral pH involves metal hydroxide precipitation. Eqs 4 and 5 were used to determine the theoretical precipitation pH (pH_ppt_). The solubility product constant, K_sp_, is the equilibrium constant used to describe the dissolution of a solid in a solution. The lower the K_sp_ of a solvation reaction, the lesser the thermodynamic favorability of dissolving that solid in a solution. More details are discussed in section 2.2.4. The study did not consider the effect of temperature on K_sp_. The value of K_sp_ at 25°C was only considered.

For dictations:

$${pH}_{ppt}=14-{log\;(\frac{C_{m}}{K_{sp}})}^{1/2}$$ (4)

For trications:

$${pH}_{ppt}=14-{log\;(\frac{C_{m}}{K_{sp}})}^{1/3}$$ (5)

Where C_m_ and K_sp_ are the metal concentration (in molarity (M)) and solubility product constant, respectively.

### 2.6. Statistical analysis

Microsoft Excel was used to perform a two-way Analysis of variance (ANOVA) double factor. The precision of the instrument obtained from ICP-OES was obtained by performing the F-test.

### 2.7. Cost analysis

All costs are expressed in US dollars (USD) considering the USD quotation of December 2019. When 1 USD was equivalent to 3.64 QR. (Qatari Riyal) [28]. To assess the cost of each test, direct and indirect costs for testing were considered. The direct cost was those related to the material used while indirect cost, including electricity and water in addition to other overhead costs.

## 3. Results and discussion

### 3.1. Physical and chemical characterizations

#### 3.1.1. Elemental analysis

Table 2 shows the results of the total acid digestion for the collected MSW-BA and the MSW-FA samples. It was revealed that Ca (1.42×10^4^ ± 2178), Fe (3.65×10^4^±548), Al (2.93×10^4^ ± 439), Mg (1.09×10^4^±163), K (6.69×10^3^±100), and Pb (52 ±1) were the major content present in MSW-BA whereas Cd (5.98 ±0.09), Ba (567± 9), Li (17.5±0.3), V (2.81 ×10^3^±42), and Sb (498 ±7) were present in the least amount. On the other hand, the MSW-FA was mostly dominated by Al (4.98×10^4^ ± 747) Ca (2.04×10^5^ ±3053), Na (1.18×10^4^ ± 620), K (1.14×10^4^± 171), Mg (1.60×10^4^ ± 240), and Fe (1.33×10^4^±200), while As (6.58 ± 0.10), Ba (696 ± 10), Mo (8.52 ± 0.18), Ni (41.5 ± 1.2), and Li (11.9 ± 0.2) were present in the least quantities. The findings were similar to Kowalski et al. [29] and Park and Heo [30] and Zhang et al. [31] The presence of such metals in the ashes can be beneficial in several ways. For instance, metal extraction from the ashes will not only reduce the consumption of raw materials but also mitigate various pollution problems that are caused due to MSW ashes disposals. Once the metals are extracted from the ashes, disposal of the treated ashes can also minimize the hazard related to leaching of metals into groundwater. The treated ashes can also be used as construction materials.

**Table 2:** Elemental compositions of the MSW-BA and the MSW-FA according to USEPA SW846 Method 3050B and Energy-dispersive X-ray spectroscopy (EDS).

Element	MSW-BA	MSW-FA
	ICP analysis—USEPA SW846 Method 3050B, (mg/L)	
Al	2.93×10^4^ ± 439	4.98×10^4^ ± 747
As	7.97 ± 0.12	6.58 ± 0.10
Ba	567 ± 9	696 ± 10
Ca	1.42×10^4^ ± 2178	2.04×10^5^ ± 3053
Cd	5.98 ± 0.09	12.8 ± 0.2
Co	21.5 ± 0.3	7.66 ± 0.11
Cr	198 ± 3	147 ± 2
Cu	1.29×10^3^ ± 19	361 ± 5
Fe	3.65×10^4^ ± 548	1.33×10^4^ ± 200
K	6.69×10^3^ ± 100	1.14×10^4^ ± 171
Li	17.5 ± 0.3	11.9 ± 0.2
Mg	1.09×10^4^ ± 63	1.60×10^4^ ± 240
Mn	574 ± 9	401 ± 6
Mo	9.88×10^3^ ± 148	8.52 ± 0.18
Na	138 ± 2	1.18×10^4^ ± 620
Ni	9.17×10^3^ ± 138	41.5 ± 1.2
P	893 ± 13	8.32×10^3^ ± 242
Pb	52 ± 1	161 ± 2
Sb	498 ± 7	99.5 ± 13.9
Sr	20 ± 1	924 ± 47
V	2.81 ×10^3^ ± 42	31.5 ± 0.2
Zn	2.93×10^4^ ± 439	4.98×10^4^ ± 747
	**Energy-dispersive X-ray spectroscopy (EDS), (wt%)**	
Al	2.80 ± 0.03	2.20±0.02
Ca	18.5±0.2	45.2±0.5
Cl	0.80±0.00	1.40±0.01
Mg	-	1.40±0.01
O	55.4±0.6	40.3±0.4
Si	22.5±0.3	9.70±0.10

Ion chromatography analysis (IC) confirmed the presence of chloride more than 160.0 ± 0.2 mg/L followed by sodium and potassium (92.4 ± 0.1 mg/L and 40.0 ± 0.0 mg/L, respectively). Moreover, the IC obtained for the MSW-FA also showed a high dominance of chloride, sodium, potassium, and sulfate (423.0 ± 0.5 mg/L, 1.76×10^5^±193 mg/L, 129.0 ±0.1 mg/L, and 19.1±0.0 mg/L, respectively). The IC confirmed that the MSW-FA had a very high concentration of chloride, almost double in contrast with the MSW-BA. High Cl and Na presence can be associated with high salt food intake, while potassium presence can be associated with high fertilizer consumption [32].

#### 3.1.2. Energy-dispersive X-ray spectroscopy (EDS)

Table 2 shows the elemental compositions of the MSW-BA and MSW-FA according to the EDS analysis. The EDS further confirmed O (55.4±0.6wt%), Si (22.5±0.3 wt%), and Ca (18.5± 0.2 wt%) as the major constitutes of MSW-BA. Moreover, the MSW-FA was found to be enriched with 45.2±0.5 wt% Ca and 40.3±0.4 wt% O. Many peaks that were not observed in EDS-spectra was probably due to the dissociation of deionized water. Both ashes were composed of amorphous alumina-silicates and lesser ammonia of the iron-rich sphere. Which is in accordance with literature data related to MSW-BA and MSW-FA Patra et al. [33], Haiying et al. [34], Pandey et al. [35] and Assi et al. [36].

#### 3.1.3. X-ray diffraction analysis (XRD)

The toxicity of MSW is not only dependent on the pollutant element concentration, but also the specification of the element [37]. Generally, the alkalinity nature of the residual ashes (MSW-BA pH of 11.33 and MSW-FA 12.25), caused the formation of hydroxide, salt, oxide, and/or carbonate components during incineration [38,39]. The chemical compositions of the MSW-BA and the MSW-FA that were analyzed using XRD showed the presence of crystalline salts, particularly K_2_O, SiO_2_, AlO_3_, Fe_2_O_3,_ and CaO in both ashes. In general, the results showed the less volatile elements such as SiO_2_ and CaO remain in the ashes due to high temperature and low volatility. Also, the presence of SiO_2_ could be due to the earth’s surface or anthropogenic activities due to the sand uptake of the waste or the erosion of the materials [40]. These results were in a good agreement with other available data from relatively similar studies including Zhang et al. [31], Bertolini et al. [37], Volokitin et al. [41], Yang et al. [42], Hussain et al. [43], Alam et al. [44], and Wongsa et al. [45].

#### 3.1.4. Bulk density, particle density, and particle size distribution (PSD)

The bulk densities of the MSW-BA and the MSW-FA were calculated by Eq 6 [24]:

$$Bulk\;Density\;(g/{cm}^{3})\frac{mass\;of\;ash}{final\;volume\;of\;ash}$$ (6)

The bulk and particle densities for the MSW-BA were 4.0 ±0.0 g/cm^3^ and 0.5±0.0 g/cm^3^, respectively, while for the MSW-FA, it was 2.5±0.0 g/cm^3^ and 0.5±0.0 g/cm^3^, respectively. The MSW-BA has a higher bulk density in contrast with the MSW-FA, almost double while the particle size density is the same for both ashes. It is important to highlight that the bulk density is inversely related to the porosity of the ash, hence the more pore space between the particles, the lower would be the bulk density, indicating low porosity. Particle density on the other hand plays an important role in understanding other physical properties. Lynn et al. [46] found that the bulk density of MSW-BA was 1386 kg/m^3^, and specific gravity/particle density was 2.3 which was lower than typical natural sand.

**Fig 2:**
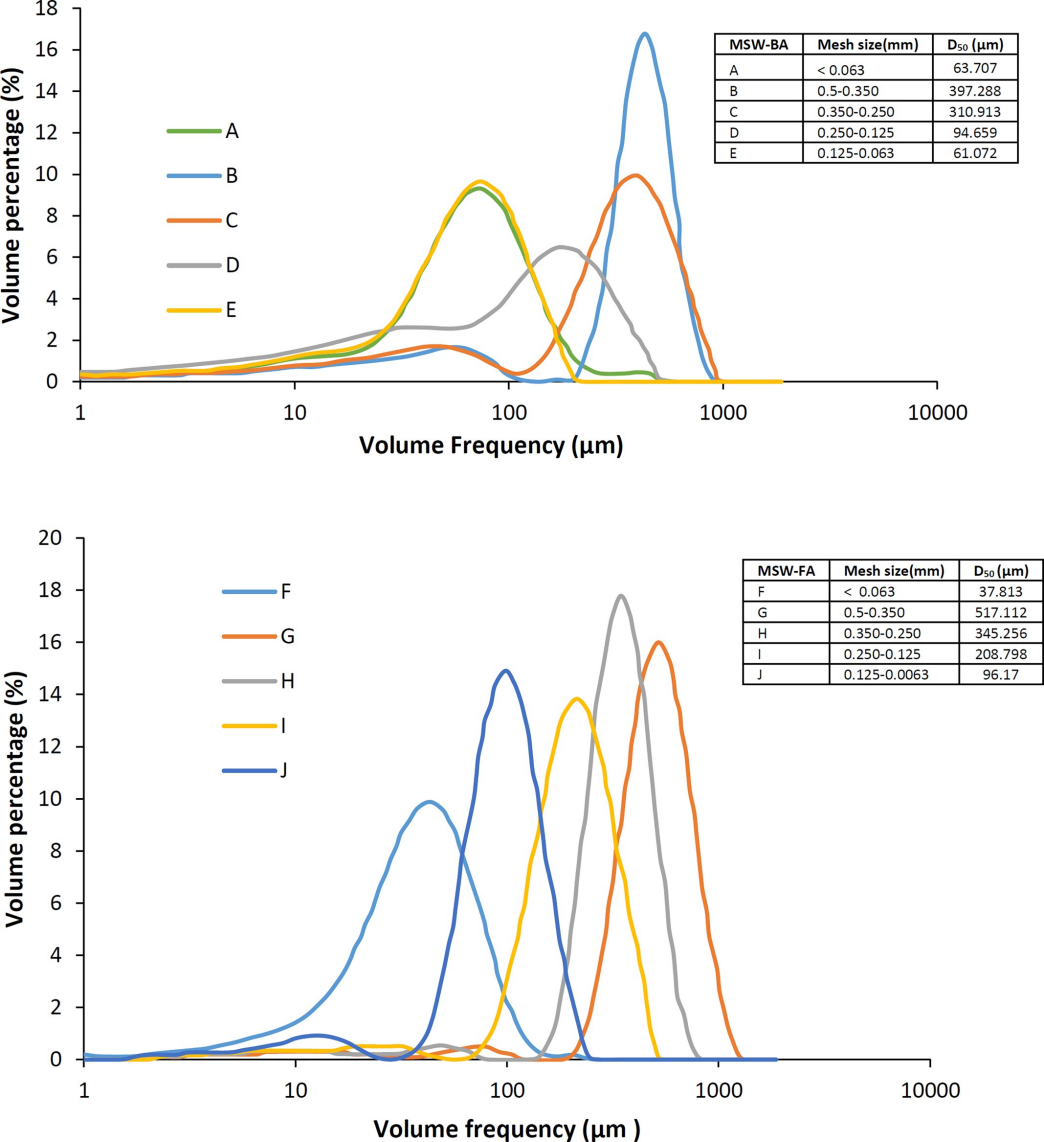
Particle size distribution of the MSW-BA and the MSW-FA using Master Sizer 3000.

Fig 2 illustrates the particle size distribution of the MSW-BA and the MSW-FA. The MSW-BA had a variety of particle size range. This behavior is expected to play a very crucial role when describing the ash kinetic behavior. For instance, when comparing the B and C graph from the D_50_ point of view, it could be said that the particle sizes are similar. However, when observing their broadness, it is evident that C is broader which indicates non-uniform particle size. This is also evident from D_w_ which was lower in B but higher for C, while from PD values, the data for B is closer to 1 which further indicates that the particles are more uniform. On the other hand, the MSW-FA was observed to be more uniform, as the peaks were narrower. This can also be further confirmed by the PD values, as it’s closer to 1. This observation is consistent with the fact that the MSW-BA is a heterogeneous material containing concrete, ceramics, glass, brick, some metals, and fused material. While MSW-FA is characterized by bimodal distribution [47]. Coarser MSW-FA is mainly formed due to fragmentation, elutriation, and various solid-vapor processes such as vaporization, nucleation, condensation, and coagulation which are believed to play a major role in the formation of finer MSW-FA [48].

#### 3.1.5. Scanning electron microscopy (SEM)

**Fig 3:**
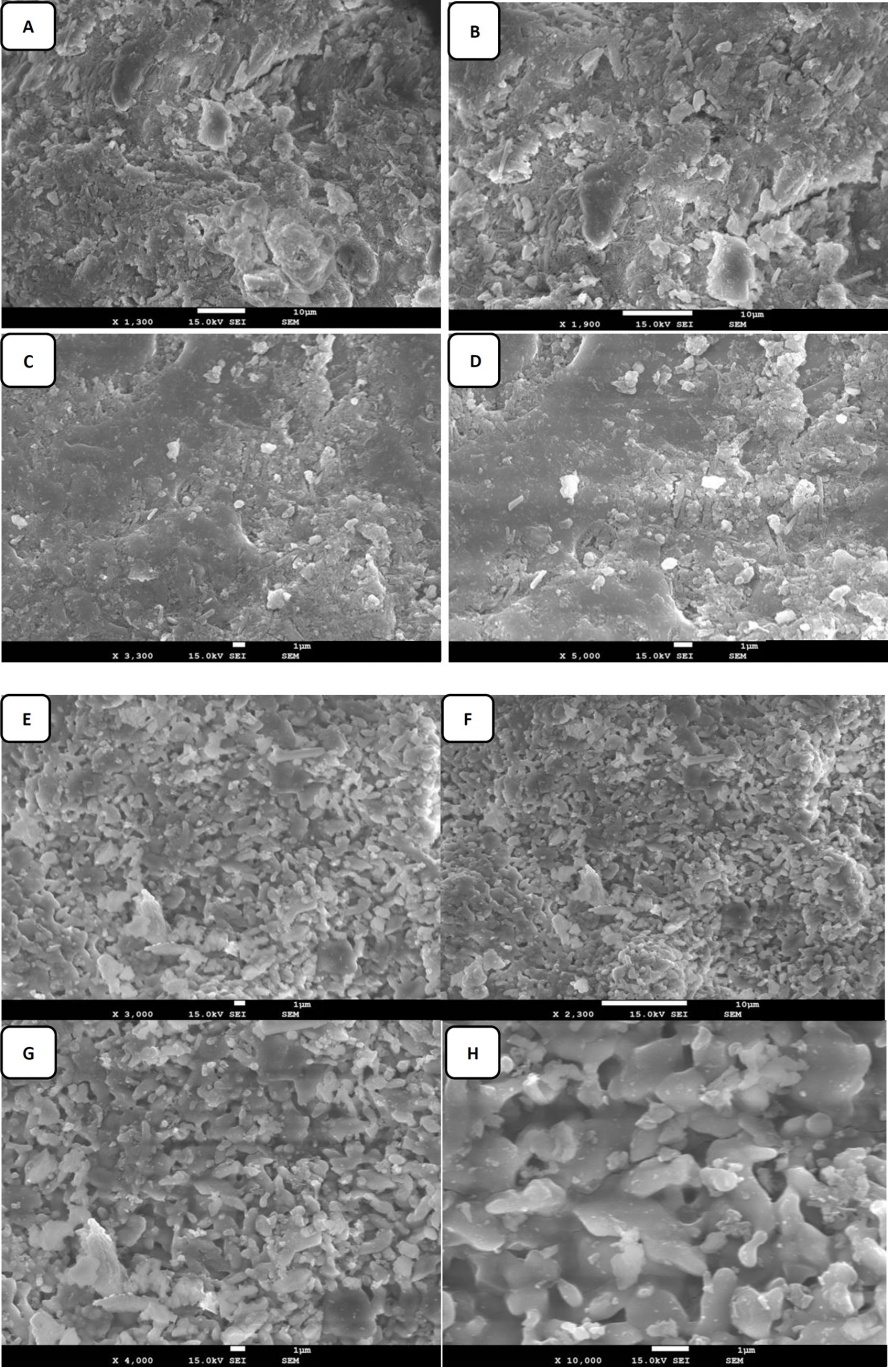
SEM images of MSW-BA and MSW-FA at different magnifications. For MSW-BA: 1300 (A), 1900 (B), 3300 (C) and 5000 (D) magnification. For MSW-FA: 3000 (E), 2300 (F), 4000 (G) and 10000 (H) magnification.

Fig 3 presents the SEM micrographs of the MSW-BA and the MSW-FA at various magnifications, which helped to understand the texture as well as the morphology of the ashes. The MSW-BA micrograph was observed at 1300, 1900, 3300, and 5000 magnification. Due to the agglomeration of the ashes, it was not possible to perform a precise quantification by surface analysis. The ash appeared as flaky and powder-like [46,49], which is generally associated with low strength and shorter dimensions (Fig 3A). While Fig 3B and 3C revealed the MSW-BA particles as irregular, angularly shaped yet closely packed together. The irregular surface texture property could be useful to prevent slipping of particles. Large pore formations that were identified, were perhaps due to the nature of the material present, which can negatively influence the mechanical strength of the MSW-BA [24]. Similar observations were made by Lynn et al. [46] and Hu et al. [49]. On the other hand, the MSW-FA micrograph was observed at 2300, 3000, 4000, and 10000 magnifications. The morphological micrographs (Fig 3E–3H) of the MSW-FA showed the presence of various agglomerated particles. The MSW-FA was observed as polycrystalline with various particle shapes and sizes. The MSW-FA was much finer and more loosely distributed. Structurally speaking, the ash was more needle, rod-shaped, and elongated (Fig 3E and 3F) [31]. It has been reported, the elongation appearance of the MSW-FA is due to the unburnt residue caused by incomplete combustion [34] MSW-FA also appeared more porous. The black spots visible in Fig 2H were probably space between particles, these spaces will most likely facilitate the leaching of metals from MSW-FA [35]. The spherical shape was commonly found in various studies [50]. Furthermore, some materials were also noticed as cuboids on the surface of the spheres, and typical porous structures were observed [51].

#### 3.1.6. Surface area, pore size distribution and density analyses

Brunauer-Emmett-Teller (BET) technique was used to study the surface area and pore size distribution of the MSW-BA and the MSW-FA. The surface area of the MSW-BA was 49.29±0.05 m^2^/g, which indicated a mesoporous particle [50]. This mainly contributes to particles that were ranging between 2–50 nm. While MSW-FA, the surface area was recorded as 2.67±0.00 m^2^/g indicating microporous. On the other hand, the pore size of the MSW-FA was 74.1 Å and for the MSW-BA was 146.2 Å. Furthermore, the isotherm acquired from both ashes showed type V, indicating small adsorbate and adsorbent interaction. Herman et al. [50] found MSW-BA as mesoporous because the pore width was 3.01 nm. While the pore volume was 0.04 cm^3^/g, the surface area was 58.01 m^2^/g, and bulk density was 2.53 g/cm^3^. While King et al. [52] investigated coal FA and found the specific surface area to be 1.39 m^2^/g. In another study, the MSW-BA surface area was reported as 17.44 m^2^/g. Fedje et al. [53] reported the specific surface area of the FA as 5.1 m^2^/g also, Hong et al. [22], found the specific area of the FA as 5.57 m^2^/g.

#### 3.1.7. Fourier Transform Infrared Spectroscopy (FTIR)

**Fig 4:**
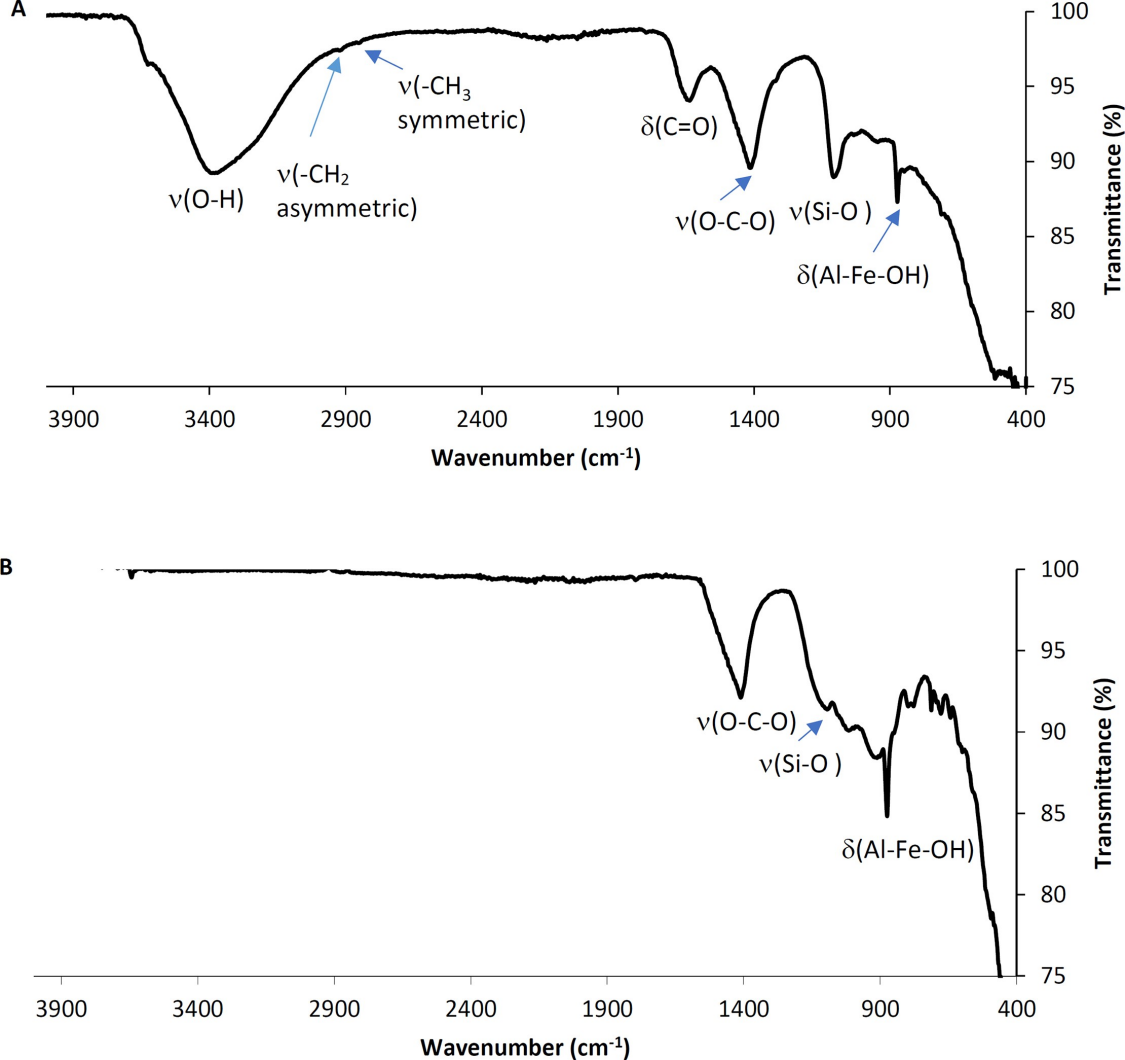
FTIR spectra of (A) MSW-BA and (B) MSW-FA.

Fig 4 presents the FTIR spectra of the MSW-BA and the MSW-FA. The characteristic broad peaks at 713 cm^-1^, 875 cm^-1^, and 936 cm^-1^ indicated the presence of Al-Fe-OH. At 1103 cm^-1^, another vibration indicated the presence of the Si-O bond and Si-O-Si stretching vibration [24,53]. A strong stretching peak was observed at 1411 cm^-1^, indicating the presence of carbonates of O-C-O [54]. Lastly, at 1636 cm^-1^, the peak signifies the presence of C = O, while a broad peak at 3400 cm^-1^ was observed, which possibly indicates hydroxyl (O-H) stretch [24]. On the other hand, the MSW-FA spectrum (Fig 4B) shows a vibration at 1103 cm^-1^ which indicates the presence of silica Si-O. The peak at 1410 cm^-1^ indicated the presence of C-O. The incineration process might have not been completed as the peaks at 2940 and 2842 cm^-1^ indicate the presence of CH_2_ asymmetric and CH_3_ symmetric stretching vibrations, respectively [55]. A broad peak was observed at 3715 cm^-1^ and 2358 cm^-1^, which represented the stretching, and deformation of OH and H-O-H groups, respectively [24]. Additionally, the prominent peaks were observed at 1640 cm^-1^ and 1450 cm^-1^ suggesting a greater contribution in water-bound and carbonates. However, the main absorption band was noted at 940 cm^-1^ that was assigned to asymmetric stretching vibration for Si-O.

### 3.4. Metal extraction

Studies have suggested that the leaching behavior of metals is dependent on various parameters including bulk properties of hosting particles, their mode of occurrence, and the pH of the leaching solution [56]. The extraction of a metal (M) using an extracting agent in an acidic solution (HA) can schematically be described as Eq 7:

$$M^{z+}+zHA↔MA_{z}+zH^{+}$$ (7)

Where HA denotes the protonated extracting agent.

When comparing coarse ash to fine particles, fine particles that have larger surface areas are exposed to leaching solution and release a greater amount of metals due to surface contact, while lower surface area promotes adsorption of metal. Nevertheless, a decrease in the particle size promotes the adsorption of metals. Major elements found in the ash include Si, Al, Ca, and Fe that affect the ash properties as well as mineralogy, which play a crucial part in the leaching of toxic metals [57,58].

**Fig 5:**
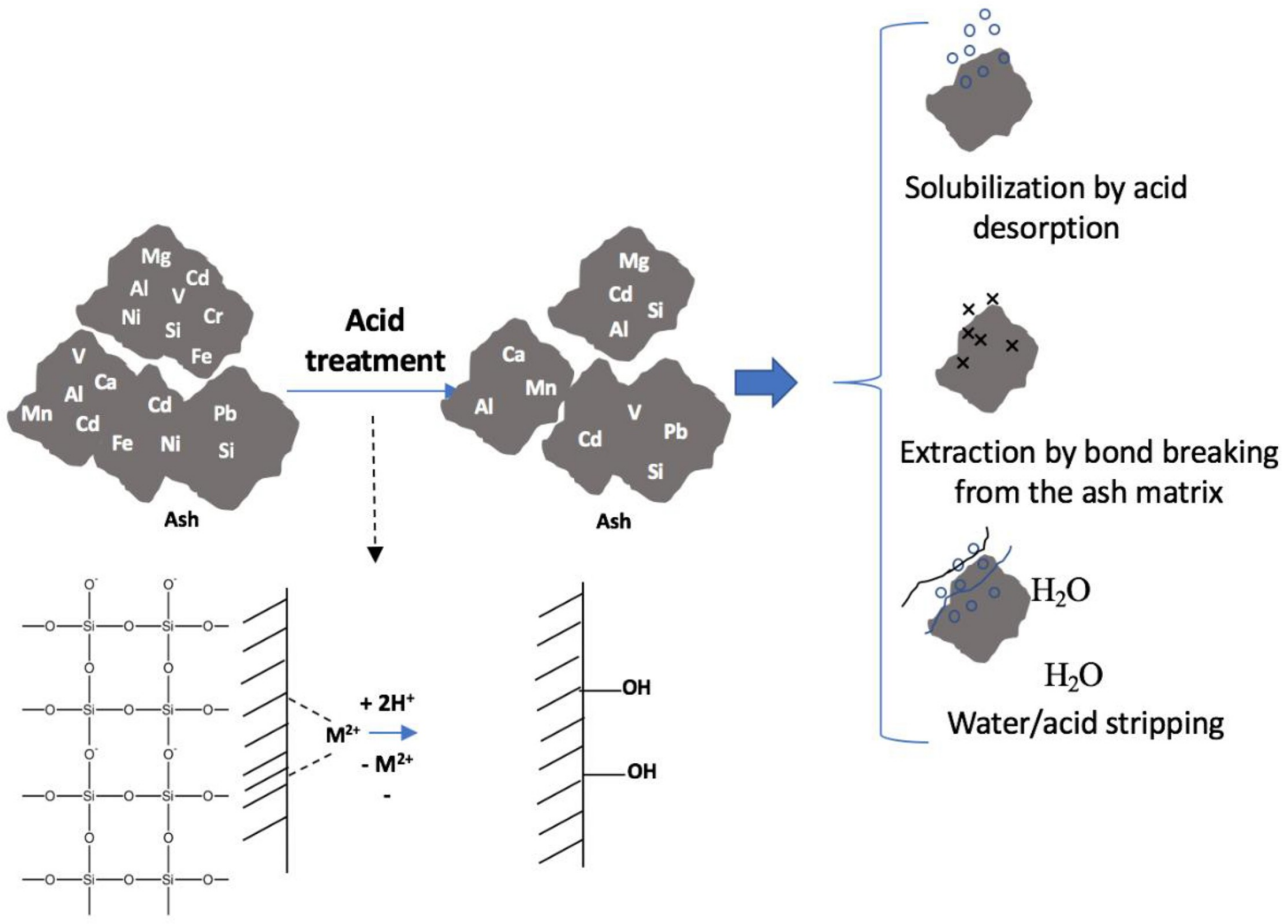
Suggested mechanisms of metal extraction from the MSW-BA and the MSW-FA.

The metal’s properties, namely electronegativity, solubility, and atomic radii affect the metals extraction and the maximum acid reachable during the ash extraction processes in addition to the metal removal kinetics. The extraction efficiency of metals from the ash could be due to different mechanisms: (i) solubilization by acid desorption, (ii) extraction by bond breaking from the ash matrix, and (iii) water/acid stripping. The electronegativity, solubility, and atomic radii of the metals are correlated with the activation energy required for the metals desorption from the ash matrix due to the affinity of the metal to the ash particle. In the extraction process, the first step would be rapid metal removal due to the effect of water/acid stripping, while, the second one is the internal diffusion phenomena. Also, the availability of the metals bound residues in the ash particle, which persist in the ash matrix, may considerably reduce the accessibility and the availability of the acid extraction process. These bound residues in the ash matrix could be described as covalently bound to the ash particle, adsorbed residues to the ash matrix by reversible non-covalent interactions; and entrapped residues, which are retained within the ash matrix. Fig 5 suggests the mechanisms of metal extraction from the MSW-BA and the MSW-FA.

#### 3.2.1. Alkaline extracting agents

The ashes by nature are basic, thus when basic solutions were used to extract metals, the extraction of the metals was almost undetectable. This could be explained due to the precipitation and/or sorption of the solution at high temperatures, making the extraction process unsuccessful. Therefore, it was concluded that the basic solutions were not feasible for the optimum extraction of metals.

#### 3.2.2. Inorganic salt extracting agents

Inorganic salt also exhibited similar results as basic extracting agents. For instance, sodium acetate, which is an inorganic salt, but when dissolved in distilled water becomes basic with a pH greater than 7. Similarly, EDTA^2-^ is a conjugate base, however, it requires basic conditions to dissolve the carboxyl group present in EDTA which makes the solution of diammonium-EDTA basic [59]. Ammonium nitrate and ammonium sulfate behave as a weak acid in aqueous solution; hence the extraction of metals was not effective.

#### 3.2.3. Acidic extracting agents

**Fig 6:**
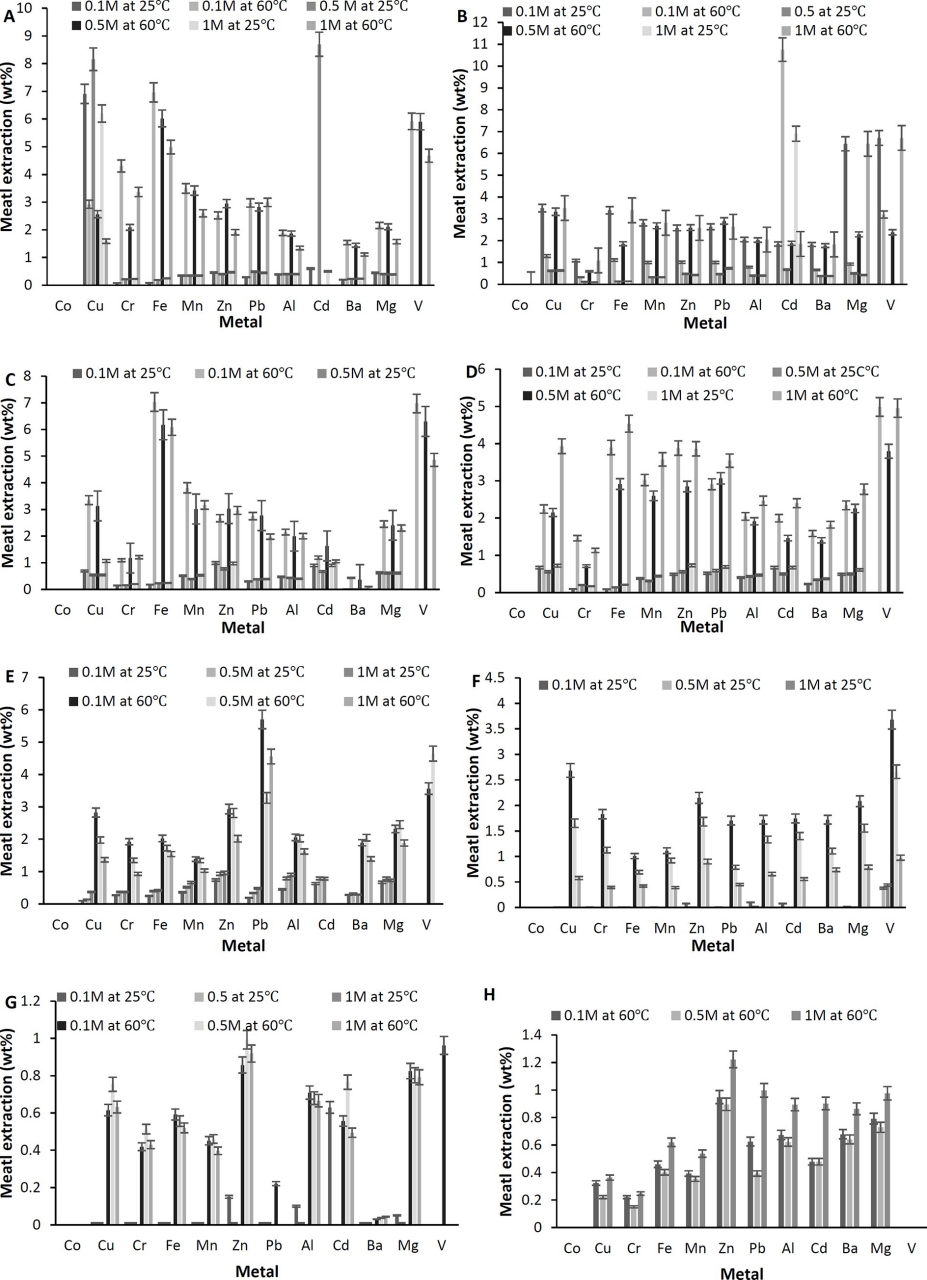
Metal extraction from the MSW-BA and the MSW-FA using acidic extracting agents. For MSW-BA: (A) Phosphoric acid, (B) Nitric acid, (C) Sulfuric acid, and (D) Perchloric acid. For MSW-FA: (E) Phosphoric acid, (F) Nitric acid, (G) Sulfuric acid, and (H) Perch.

Under the acidic environment, the metals were drastically leached in contrast with the alkaline solution. This can perhaps be explained due to the neutralization process followed by the disassociation of oxides and carbonates which were formed earlier during the incineration process as discussed earlier. A redox reaction might also have played a vital role in releasing some of the metals in the aqueous solution. As observed from XRD various meals were present in their mineral forms such as K_2_O, SiO_2_, AlO_3_, Fe_2_O_3,_ and CaO. Disassociation of such minerals in a reducing environment will most likely lead to the release of the desired metals. The results are expressed in terms of the percentage of metal extraction using various acids i.e. perchloric acid, phosphoric acid, sulfuric acid, and nitric acid. Various concentrations of the previously-mentioned acids were tested as well as the effect of temperature. Fig 6A–6D display the metal percentage extracted from the MSW-BA using phosphoric acid, nitric acid, sulfuric acid, and perchloric acid, respectively. Fig 6E–6H show the percentage of the metal extracted from the MSW-FA using the same acid as the MSW-BA. From all treatments, it was concluded Co was unable to be extracted by any treatment from both ashes. From MSW-BA, phosphoric acid could extract 6.91wt% of Cu and 4.3 wt% of Cr at 25°C and 60°C, respectively. At a high temperature, around 7 wt% of iron and 3.4 wt% of Mn was extracted using sulfuric acid and phosphoric acid, respectively. Ba, Pb, and Al in general were observed to yield high percentage removal at higher temperatures (1.8 wt%, 3.5 wt%, and 2 wt%). The highest percentage of V was observed with both acids, sulfuric acid, and phosphoric acid at 60°C, yielding up to (7 wt% and 6 wt%, respectively).

On the other hand, the MSW-FA showed a similar trend; however, it was observed in almost all the metals, a high temperature yielded the highest percentage removal. Cu, Cr, Fe, Zn, and Pb gave the highest percentage using phosphoric acid (3 wt%, 2 wt%, 2 wt%, 3 wt%, and 5.6 wt%, respectively) at 60°C. A 4.6 wt% of V was extracted using phosphoric acid at 0.5 M and 60˚C. It is important to highlight one of the reasons why perhaps the MSW-FA had the lower percentage removal in contrast with the MSW-BA; it could be due to the initial high concentration of metals present in the raw ashes. It was observed that in some cases, it was easier to extract some metals from the MSW-BA while in other cases; it was easier to extract from the MSW-FA. This perhaps can also be associated with the chemical bonding between ashes and the metal. Some metal binding was so strong, it was impossible to disassociate the metals while in some cases the bonds were weak. Al-Ghouti et al. [60] mentioned that increasing the amount of the extraction agent facilitates the reaction. It was observed that the highest percentage removal of Cu, Cr, Fe, Al, V were obtained by using 0.5 M phosphoric acid at 25°C, while Cr and Fe in 0.1 M at 60°C, Al, and V were obtained using 0.5 M at 60°C. However, it was also found V could also be extracted using a low concentration of acids. Al was extracted using nitric acid. Mn, Zn and Ba gave good results when interacted with 0.1 M and 1 M of perchloric acid at 60°C, 0.5 M at 25°C. Huang et al. [61] partially digested ashes using HNO_3_/H_2_O_2_/HF acids. The water-soluble dilatation was introduced which was expected to collect some metals including Ca, Pb, Na, and K but it was found 20 6 of MgCl_2_ was required to separate the metals.

The extraction of metals from the ash is very complicated as different metals reacting differently with the extraction agent, which gives different efficiency rate. Studies suggest various techniques that were found successful in other regions. Though there are various other methods applied in other studies that utilize various complex techniques to extract metals, however, this paper explored different options; aiming to outline a method to successfully extract maximum metals from the ashes. The results were compared with few studies, which explored the possibility to extract metals [53,56].

Analysis of variance (ANOVA) was used to validate the data and understand the effect of concentration on the metal extraction. If the F-value is greater or equal to F-critical and P-value is less than or equal to 0.05, then the data is significantly different amongst the mean of groups (Mahmoud et al., 2014) [62]. The results from Table 3 illustrate that metal extraction from the MSW-BA and the MSW-FA was significantly affected by the acid type as indicated by the F-value that is greater than F-critical while the P-value is less than 0.05.

**Table 3:** Analysis of variance (ANOVA) of the metal extraction for the MSW-BA and the MSW-FA.

**MSW-BA**			
**Acid**	**F-value**	**P-value**	**F-critical**
**Phosphoric acid**	1.712	8.475×10^−13^	1.538
**Nitric acid**	14.05	7.414×10^−32^	1.538
**Sulphuric acid**	540.0	5.548×10^−138^	1.538
**Perchloric acid**	51.16	3.611×10^−98^	1.538
**MSW-FA**			
**Phosphoric acid**	32.94	2.227×10^−77^	1.550
**Nitric acid**	2201	1.191×10^−128^	1.538
**Sulphuric acid**	315.2	1.232×10^−104^	1.530
**Perchloric acid**	130.1	1.079×10^−107^	1.530

#### 3.2.4. Solubility product constant for hydroxide and carbonate

Table 4 shows the hydroxide and carbonate K_sp_ values adapted from Lide (2006) [63] and precipitation values of the extracted metals from the MSW-BA and the MSW-FA. Precipitation determination was performed based on the assumption that all ions present, are in their most stable ionic state. For instance, aluminum would be first precipitated because as indicated in Table 4, at pH 3.56, Al precipitated before any other metals followed by Cr and Cu. Furthermore, from the K_sp_, it can be assumed that in general for both MSW-BA and MSW-FA, PbCO_3_ will precipitate before BaCO_3_ as K_sp_ for the former is lower than the latter (7.4×10^−14^ and 5.0×10^−9^, respectively). On the other hand, in the MSW-FA, Al would also precipitate first at pH 3.56 followed by Cr, Cu, and Zn. From Table 4, it can be concluded that the pH_ppt_ of metals varies from one metal to another. Based on the data given in Table 4 in the MSW-BA, Zn will precipitate at pH 6.93, Ba at pH 8, Pb at pH 8.45 Cd at pH 9.12, Fe at pH 9.56, Mn at pH 11.45 and Mg at pH 11.72. While for the MSW-FA as indicated from Table 4, Pb will precipitate at pH 8.72 followed by Fe at pH 9.74, Cu at pH 9.80, and Mg at pH 11.12. Since Al_2_(CO_3_)_3_ is soluble in solution and; therefore has no K_sp_ likewise Cr_2_(CO_3_)_3_.

**Table 4:** K_sp_ values and pH of precipitation for the extracted metals for the MSW-BA and MSW-FA (the K_sp_ values were adapted from solubility product contstant [63]).

Metal	Extracted concentration^a^ (C_m_, mol/L)	Best extracting agent	Hydroxide		Metal	Carbonate	
			K_sp_ (as hydroxides at 25°C)	pH_ppt_ (precipitation)^b^		K_sp_ (as carbonates at 25°C)	pH_ppt_ (precipitation)^b^
**MSW-BA**							
Al^3+^	2.87×10^−4^	Sulfuric acid	1.9×10^−33^	3.62	Pb^2+^	7.4×10^−14^	9.36
Cr^3+^	1.6×10^−4^	Phosphoric acid	1.6 10^−30^	4.3	Zn^2+^	1.4×10^−11^	9.96
Cu^2+^	7.07×10^−4^	Nitric acid	2.2 ×10^−20^	5.72	Cd^2+^	1.0×10^−12^	10.19
Zn^2+^	1.6×10^−3^	Sulfuric acid	1.2×10^−17^	6.93	Mn^2+^	1.8×10^−11^	10.45
Ba^2+^	5×10^−3^	Nitric acid	5×10^−3^	8.00	Cu^2+^	1.4×10^−10^	10.62
Pb^2+^	1.32×10^−4^	Perchloric acid	1.2×10^−5^	8.45	Ba^2+^	5.1×10^−9^	10.92
Cd^2+^	4.15×10^−5^	Sulfuric acid	7.2×10^−15^	9.12	Fe^3+^	3.2×10^−11^	11.09
Fe^3+^	1.66×10^−2^	Phosphoric acid	8.0×10^−16^	9.56	Mg^2+^	3.5×10^−8^	11.72
Mn^2+^	2.55×10^−4^	Phosphoric acid	1.9×10^−9^	11.46			
Mg^2+^	1.24×10^−2^	Perchloric acid	3.5×10^−8^	11.72			
**MSW-FA**							
Al^3+^	3.8×10^−2^	Phosphoric acid	1.9×10^−33^	3.56	Cr^3+^	5.1×10^−9^	4.32
Cr^3+^	1.82×10^−4^	Phosphoric acid	1.6 × 10^−30^	4.32	Pb^2+^	1.8×10^−11^	8.72
Cu^2+^	1.62×10^−4^	Phosphoric acid	2.2 ×10^−20^	6.07	Zn^2+^	3.5×10^−8^	10.14
Zn^2+^	7.3×10^−4^	Phosphoric acid	1.2 ×10^−17^	7.10	Mn^2+^	1.4×10^−10^	10.56
Pb^2+^	4.43×10^−5^	Phosphoric acid	1.2 ×10^−5^	8.72	Cd^2+^	1×10^−12^	10.87
Fe^3+^	4.82×10^−3^	Phosphoric acid	8.0×10^−16^	9.74	Cu^2+^	1.0×10^−12^	10.97
Cd^2+^	1.98×10^−6^	Sulfuric acid	7.2×10^−15^	9.80	Mg^2+^	3.2×10^−11^	11.17
Mg^2+^	1.61×10^−2^	Phosphoric acid	3.5×10^−8^	11.12	Fe^3+^	3.20×10^−11^	11.27
Mn^2+^	1.45×10^−4^	Phosphoric acid	1.9×10^−9^	11.57			
Ba^2+^	1.01×10^−4^	Phosphoric acid	5×10^−3^	14.0			

^a^ (experimental conditions):

^b^For dications: pH_ppt_ = 14—log(C_m_/K_sp_)^1/2^ and for trications: pH_ppt_ = 14—log(C_m_/K_sp_)^1/3^; where C_m_ and K_sp_ are the metal content (in molarity (M)) and solubility product constant, respectively.

### 3.3. Cost analysis

The cost of metal extraction from ash is a key component in promoting and application of the proposed extraction procedure. It is a common method used in decision making and predicting the possible environmental effects in the overall application process. The cost needs to cover operating expenses, including chemical and consumable cost, multiple iterative assessments, batch process optimization, and the number of experiments, electricity cost, and others. The complete process of the metal extraction was carried out in a lab-scale. Hence, the cost analysis was performed considering lab-scale metal extraction.

To propose the cost analysis, a detailed analysis was established by including the following factors, the cost of the acid material, energy consumption, miscellaneous expenses, and other laboratory efforts to treat a given amount of ash. This model allowed for different sizes of the batch process to be analyzed for overall efficiency and cost that could treat 1 kg of ash input.

For the metal extraction, it was concluded that nitric acid and phosphoric acid were the best-suited acids for the MSW-BA while sulfuric acid and phosphoric acid for the MSW-FA. Based on this conclusion, the breakdown of the cost of each step of the metal extraction process for the MSW-BA and the MSW-FA is presented in Table 5. The calculation was also based on the metal extraction at 0.1 M and 60°C.

**Table 5:** Breakup and total cost required of metal extraction for 1 kg of the MSW-BA and the MSW-FA.

No.	Item	Unit cost, USD	Amount used	Cost, USD
**MSW-BA**				
**1**	Nitric acid (ACS reagent, 2.5 L)	32.9	0.447 L	5.88
**2**	Phosphoric acid (ACS reagent, 2.5 L)	35.6	0.583 L	8.30
	Cost of ash sieving and drying	0.036 per kWh	8.40 kWh (100°C; 24 h), (Energy to Heat at 100°C = 0.35 kWh/Hr)	0.302
**5**	Cost of heating	0.036 per kWh	7.44 kWh (60°C; 24 h), (Energy to Heat at 60°C = 0.31 kWh/Hr)	0.268
**7**	*Net cost*			14.8
**8**	Other overhead costs (10% of the net cost)			1.48
	**Total cost**			**16.3**
**MSW-FA**				
**1**	Sulphuric acid (ACS reagent, 2.5 L)	38.4	0.533 L	8.19
**2**	Phosphoric acid (ACS reagent, 2.5 L)	35.6	0.583 L	8.30
	Cost of ash sieving and drying	0.036 per kWh	8.40 kWh (100°C; 24 h), (Energy to Heat at 100°C = 0.35 kWh/Hr)	0.302
**5**	Cost of heating	0.036 per kWh	7.44 kWh (60°C; 24 h), (Energy to Heat at 60°C = 0.31 kWh/Hr)	0.268
**7**	*Net cost*			17.1
**8**	Other overhead costs (10% of the net cost)			1.71
	**Total cost**			**18.8**

## 4. Conclusion

The present study aimed to investigate the physicochemical characteristics and potential of metal extraction from MSW-BA and MSW-FA that are generated from one of the incineration plants in Qatar. Morphologically, the MSW-BA was heterogeneous, flaky, and powdery indicating low strength. However, the images also revealed that the MSW-BA was rough, irregular, and angularly shaped yet closely packed together. On the other hand, the MSW-FA was observed to be looser and finely distributed as evident from their structure, the ashes were more rode shaped and elongated. Both ashes were found rich with various metals including Fe, Al, Mg, and Pb in MSW-BA while Al, Ca, Na, K, Mg, and Fe in MSW-FA. Furthermore, K_2_O SiO_2_ AlO_3_ Fe_2_O_3_, CaO were amongst the major crystals present in both ashes. Particle size distribution revealed that the MSW-BA was heterogeneous with a variety of particle size distributions, while the MSW-FA was found to be more towards bimodal distribution. Al-Fe-OH, Al-Al-OH, Si-O, C-O, and C-H were amongst some of the major functional groups found in both ashes.

It was found that both ashes preferred acidic solutions as a high leading to a high percentage of metals being extracted from the MSW-BA and the MSW-FA. The study also explored the possibility to extract various metals from the MSW-BA and the MSW-FA using various extraction agents. This study found the acid solutions were effective for metal extraction. More than 11 wt% of Cd and 9 wt% of Cu were extracted from the MSW-BA, while 6 wt% of Pb 4.5 wt% of V was extracted from the MSW-FA with only 6 h contact time. The overall extraction yield of metals in aqueous phases can be used for further metal extraction using a leaching-extraction procedure with a longer duration.

Furthermore, the metal extraction from the MSW can be an effective way to recycle the ashes. The present technique is an interesting development in metal extraction from MSW-BA and MSW-FA, which can develop in a cost-effective and sustainable option to utilize Qatar MSW. However, further studies are required for the upscaling of the solvent leaching technique to obtain maximum metal extraction with minimum energy utilization. This work can contribute to future work involving the utilization and application of problematic MSW-BA and MSW-FA.

## Supporting information

S1 Graphical abstract (TIF)

## Abbreviations

ANOVA: Analysis of variance
BA: Bottom Ash
BCR: Bureau of Reference
BET: Brunauer Emmett Teller
$\bar{D_{n}}$: Number-average diameter
$\bar{D_{w}}$: Weight-average diameter
FA: Fly Ash
EDX: Energy-Dispersive X-Ray Spectroscopy
FTIR: Fourier-Transform Infrared Spectroscopy
ICP-OES: Inductively Coupled Plasma—Optical Emission Spectrometry
IC: Ion Chromatography
GCC: Gulf Cooperation Council
K_sp_: Solubility Product
MSW: Municipal Solid Waste
MSW-BA: Municipal Solid Waste—Bottom Ash
MSW-FA: Municipal Solid Waste—Fly Ash
N_i_: Number of particles at the *i* th class in the size-distribution histogram
pH_ppt_: Precipitation pH
SEM: Scanning Electron Microscopy
XRD: X-Ray Diffraction
