# Supplementary figures and images for: Physiochemical characterization and systematic investigation of metals extraction from fly and bottom ashes produced from municipal solid waste
Source: PLoS One. 2020 Oct 22;15(10):e0239412. doi: 10.1371/journal.pone.0239412 (PMC7580925; doi:10.1371/journal.pone.0239412)

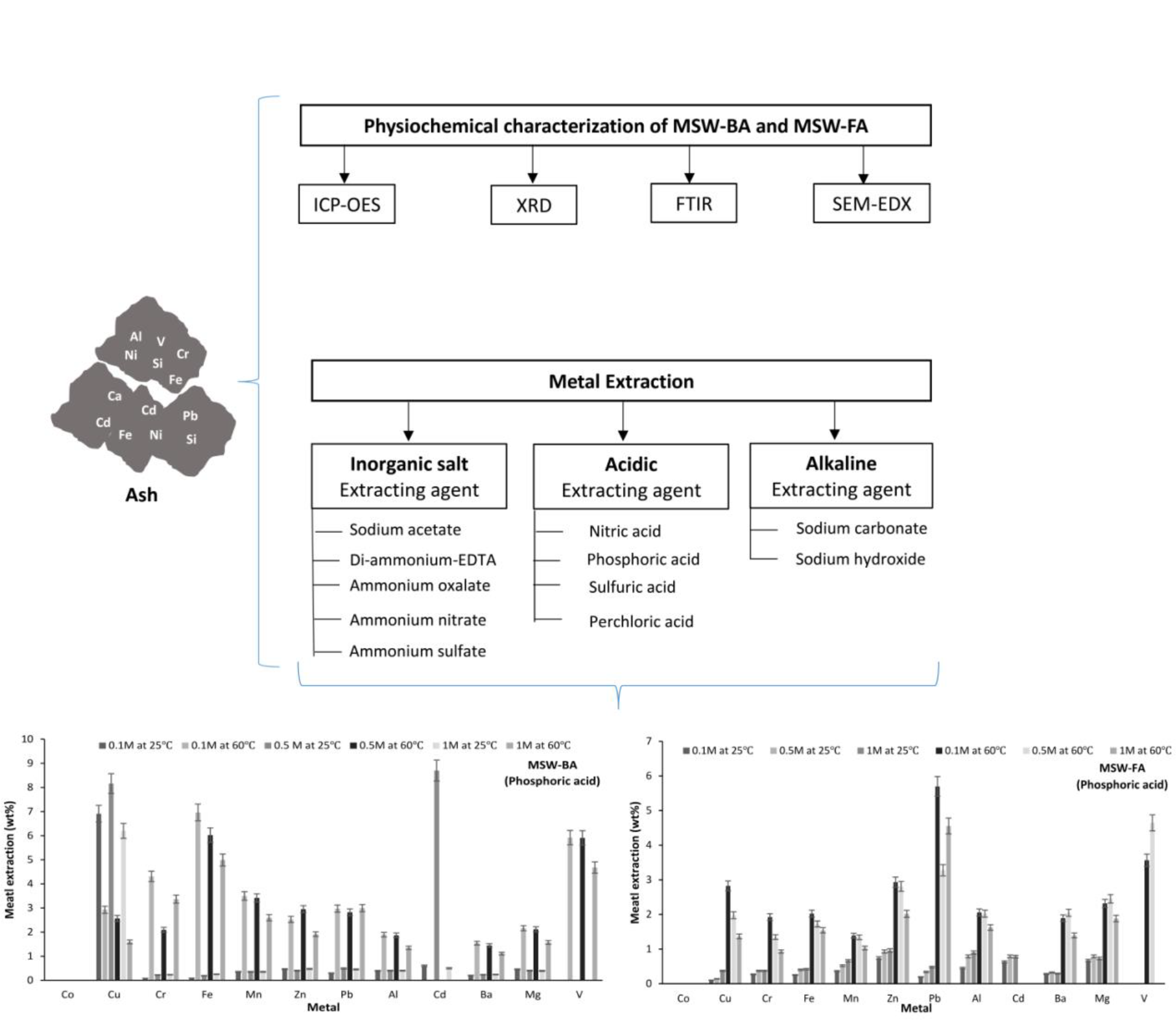

Supplement: S1 Graphical abstract — (TIF) [file pone.0239412.s001.tif]
